# Antimicrobial stewardship in paediatrics

**DOI:** 10.1186/s12879-016-1772-z

**Published:** 2016-08-18

**Authors:** Nicola Principi, Susanna Esposito

**Affiliations:** Department of Pathophysiology and Transplantation, Pediatric Highly Intensive Care Unit, Università degli Studi di Milano, Fondazione IRCCS Ca’ Granda Ospedale Maggiore Policlinico, Via Commenda 9, Milan, 20122 Italy

**Keywords:** Antibiotics, Antimicrobial resistance, Antimicrobial stewardship, Bacterial resistance

## Abstract

**Background:**

Antibiotics are among the drugs most commonly prescribed to children in hospitals and communities. Unfortunately, a great number of these prescriptions are unnecessary or inappropriate. Antibiotic abuse and misuse have several negative consequences, including drug-related adverse events, the emergence of multidrug resistant bacterial pathogens, the development of *Clostridium difficile* infection, the negative impact on microbiota, and undertreatment risks. In this paper, the principle of and strategies for paediatric antimicrobial stewardship (AS) programs, the effects of AS interventions and the common barriers to development and implementation of AS programs are discussed.

**Discussion:**

Over the last few years, there have been significant shortages in the development and availability of new antibiotics; therefore, the implementation of strategies to preserve the activity of existing antimicrobial agents has become an urgent public health priority. AS is one such approach. The need for formal AS programs in paediatrics was officially recognized only recently, considering the widespread use of antibiotics in children and the different antimicrobial resistance patterns that these subjects exhibit in comparison to adult and elderly patients. However, not all problems related to the implementation of AS programs among paediatric patients are solved. The most important remaining problems involve educating paediatricians, creating a multidisciplinary interprofessional AS team able to prepare guidelines, monitoring antibiotic prescriptions and defining corrective measures, and the availability of administrative consensuses with adequate financial support. Additionally, the problem of optimizing the duration of AS programs remains unsolved. Further studies are needed to solve the above mentioned problems.

**Conclusions:**

In paediatric patients, as in adults, the successful implementation of AS strategies has had a significant impact on reducing targeted- and nontargeted-antimicrobial use by improving the quality of care for hospitalized patients and preventing the emergence of resistance. Considering that rationalization of antibiotic misuse and abuse is the basis for reducing emergence of bacterial resistance and several clinical problems, all efforts must be made to develop multidisciplinary paediatric AS programs in hospital and community settings.

## Background

Antibiotics are among the drugs most commonly prescribed to children in hospital and community settings [1–3]. It has been reported that the average proportion of children in hospital settings who receive at least one antibiotic is between 33 % and 78 % [4–8]. Moreover, antibiotics are prescribed during approximately 20 % of paediatric ambulatory visits [9]. Unfortunately, a great number of these antibiotic prescriptions are unnecessary or inappropriate. Frequently, antibiotics are administered to children who suffer from viral infections or from non-infectious diseases [10–12]. In other cases, broad-spectrum antibiotics are given to children who suffer from infections for which narrow-spectrum drugs are indicated and recommended [9, 11]. Finally, many children receive antibiotic prescriptions indicating an incorrect total daily dosage or fractioning or for a period of time significantly longer than needed [4, 10–14].

Antibiotic abuse and misuse have several negative consequences. The incidence of drug-related adverse events is significantly increased in instances of improper antibiotic use [15, 16]. Moreover, the emergence of multidrug resistant bacterial pathogens is heavily favoured in such instances, leading to longer hospital stays, increased patient mortality and increased healthcare costs [15, 16]. Other important problems related to antibiotic abuse and misuse are represented by the increasing incidence in *Clostridium difficile* infection [17, 18] and the negative impact on microbiota [19, 20]. Inappropriate dosage may also be associated with undertreatment risks [21].

In the last 15 years, significant deficiencies have occurred in the development and availability of new antibiotics able to combat instances of emerging resistance [22]; therefore, the implementation of strategies to preserve the activity of existing antimicrobial agents has become an urgent public health priority. Antimicrobial stewardship (AS) is one such approach. In this paper, the principle of and strategies for paediatric AS programs, the effects of AS interventions and the common barriers to development and implementation of AS programs are discussed.

## Discussion

### Principle of and strategies for antimicrobial stewardship programs in paediatrics

For several years, AS efforts were focused on adult populations. A very successful and evidence-based AS program in adults was the Transatlantic Taskforce on Antimicrobial Resistance, established by American President Barack Obama and President of the European Union (EU) Fredrik Reinfeldt from Sweden [23]. Recommendations fell into three broad categories: urgent antimicrobial resistance issues focused on appropriate therapeutic use of antimicrobial drugs in the medical and veterinary communities, prevention of both healthcare- and community-associated drug-resistant infections, and strategies for improving the pipeline of new antimicrobial drugs.

The need for formal AS programs in paediatrics was officially recognized only recently, considering the widespread use of antibiotics in children and the different antimicrobial resistance patterns that these subjects exhibit in comparison to adult and elderly patients [24]. In the United States, the Infectious Disease Society of America (IDSA) published guidelines for developing an institutional program to enhance AS and identified paediatrics as a priority area for further research regarding the effectiveness of AS activities [25]. Later, the Pediatric Infectious Diseases Society in 2010 [26] and the American Academy of Pediatrics in 2012 [27] both highlighted the importance of AS in paediatrics; these organizations promoted research, developed educational programs, and recommended implementation of AS programs in health care organizations that provide inpatients and outpatients with paediatric care. After these recommendations, the number of hospitals in which AS programs have been developed and tested significantly grew. In a study recently carried out in the United States, it was found that 31 out of 42 children’s hospitals that are members of the Children's Hospital Association had a formal AS program or were in the process of implementing a program [27]. Moreover, some attempts to introduce AS in ambulatory setting have been made [28].

From a theoretical point of view, to be maximally effective in rationing antibiotic prescriptions, AS strategies and protocols must account for several elements strictly related to the antibiotic prescription but should also consider the need for continuous access to expertise in clinical pharmacology and infectious diseases and for transparent monitoring of antibiotic use (Table 1). Practically, two main core strategies are utilized, which are a prospective audit with feedback and a prior approval strategy. In the first strategy, reviews of prescriptions with feedback on antibiotics used are performed after the antibiotics have been prescribed. In the second, reviews and approvals of antibiotic prescriptions are made prior to the initiation of therapy [24]. Additional possibilities to supplement core strategies or to improve antibiotic prescribing methods include education of providers, guidelines, streamlining/de-escalation therapy, intravenous-oral conversion, dose optimization, and use of antimicrobial order forms [26, 28]. Educational programs are particularly important [26]. They should provide adequate information about the rules for the identification of patients for whom antibiotics are necessary, the optimal timing of drug administration and the most appropriate antibiotic regimen with the time of de-escalation or discontinuation specified. Goldman et al. retrospectively studied antibiotic prescriptions made over 5 years in a paediatric hospital in the United States and found that community-acquired pneumonia and ear/nose/throat infections were the diagnoses with the highest predictive probability of warranting an AS program recommendation, whereas fever/neutropenia diagnoses had the lowest probability [29]. Though single strategies can be effective, the best results with the highest reduction in antibiotic misuse are usually obtained when combined instead of single-strategy approaches are used [30].

**Table 1:** Antimicrobial stewardship strategies in paediatric settings

Main Strategies Review and analyse antibiotic use after they have been prescribed Reach consensus on antibiotic use before they are prescribed Problems that must be considered for rational antibiotic use Prompt initiation of antibiotic use when indicated Avoiding use of antibiotics for conditions not due to bacteria Choice of the first and second line drugs for the demonstrated or supposed bacterial etiology responsible for the disease that requires treatment Identification of proper dose, fractioning, and duration of antibiotic and switch from intravenous to per os according to the patient and the disease Choice of conditions for which antibiotic prophylaxis is needed Methods to rationalize antibiotic therapy Education (i.e., lectures, handbooks, educational conferences, guidelines) Use of antibiotic order forms Formation of multidisciplinary antimicrobial stewardship team Obtaining administrative and leadership support Continuous and transparent monitoring of antibiotic use Adequate use of diagnostic tests, including point-of-care tests Knowledge of local resistance rates for different pathogens	

With regards to the problem of selection of the appropriate antibiotic regimen for each child, it must be considered that for many paediatric infectious diseases, one of a limited number of bacterial pathogens is usually the etiologic cause and that, lacking direct microbiological evidence, the antibiotic of choice to treat each disease is the one active against the most probable infectious agent. To solve the problem of the delay with which culture results indicate the etiology of a given disease, the use of rapid diagnostic tests must be considered [31]. Moreover, a thorough understanding of the local rates of resistance to different pathogens is essential, and an automated update of empiric antimicrobial prescription guidelines must be planned [28, 32, 33].

The questions of the appropriate dosage and timing of antibiotic administration can be resolved by restricting formulary for empiric treatment to 48–72 h to permit a re-evaluation of the prescribed therapy and a decision as to whether the therapy should be continued, modified or suspended. However, in planning AS programs, particular attention must be paid to drugs that have been found to be predictive of receiving paediatric AS recommendations. In their study, Goldman et al. reported that third-generation cephalosporins and clindamycin were the antimicrobials with the highest predictive probability of warranting an AS program recommendation, whereas linezolid had the lowest probability [29]. Moreover, in children receiving antibiotics frequently associated with severe adverse events, monitoring of drug levels can be an opportunity to assure adequate treatment [26].

Development of local AS teams including experts from different fields is considered essential to ensure adequate development of AS programs and continuously updated information useful for avoiding antibiotic misuse and abuse [26]. Infectious disease, pharmacy, microbiology, infection control, and information technology specialists need to be involved and offer support and continuous medical education to paediatricians regarding the prescription of antibiotics. The hospital administration and medical staff leadership must also contribute, even with financial support, to the application of AS programs [26].

Implementation of paediatric AS programs is easier in the hospital setting, where technical and human resources are more plentiful and easily available than in the community. Hyun et al. have listed potential strategies to promote AS programs in community-based settings, highlighting the need for identification of local physician champions and potential resources (i.e., pharmacy records, infection control practitioners, pharmacists, microbiologists, microbiology results, information technology) as well as the great difficulties in developing common guidelines for antibiotic use and control [34].

Although AS programs should account for all the aspects previously discussed, in most previously implemented programs, the development and implementation were only partial. A relevant example is provided by the study published by Bryant on behalf of the Australasian Stewardship of Antimicrobials in Paediatrics group [35]. Fourteen tertiary paediatric hospitals were surveyed, and it was found that all of them had empirical guidelines for prescribing antimicrobials. However, most of the hospitals did not have guidelines for antifungal prophylaxis, surgical prophylaxis, neonatology or paediatric intensive care. All hospitals had restricted drugs, but only four had electronic approval systems. Auditing methods varied widely but were mostly ad hoc, with feedback on results given in an untargeted way. There was a paucity of AS education: no hospitals provided education for senior medical staff, and four had no education for any staff.

### Impact of antimicrobial stewardship programs in paediatrics

Several evaluations of paediatric AS programs have shown favourable outcomes, including a reduction in antibiotic prescriptions and lower costs (Table 2). Most of these evaluations were performed in hospitals, probably due to the difficulties in performing AS outside this setting. Among the studies concerning hospitalized children, those by Agwu et al. [36], Metjian et al. [37], and Di Pentima et al. [38] deserve attention.

**Table 2:** Main studies on the impact of antimicrobial stewardship programs in paediatric settings

Author and year of publication	Site of evaluation	Strategy	Type of control	Main results
Agwu et al., 2008 [36]	Single hospital	World Wide Web-based antimicrobial restriction program, automated clinical decision support, facilitated approval, enhanced real-time communication among prescribers, pharmacists, and paediatric infectious disease fellows	Before and after implementation of the program, evaluation of user satisfaction, reports of missed and/or delayed doses, antimicrobial dispensing times and costs	Satisfaction increased from 22–68 % and from 13–69 % among prescribers and pharmacists, respectively. Reductions of 21 % and 32 % in the number of missed and delayed antimicrobial doses, respectively $370,069 reduction in projected annual costs
Metjian et al., 2008 [37]	Single hospital	Evaluation of outcomes and compliance resulting from empirical antibiotic therapy decisions	Intervention to modify antibiotic therapy	45 % of prescriptions required an intervention.
Di Pentima et al., 2011 [38]	Single hospital	Antimicrobial use indications were included as a mandatory field in the computerized information system	Prescriptions were reviewed by specialists	Reduction in antimicrobials of more than 30 % for both targeted and nontargeted drugs
Hersh et al., 2015 [39]	Nine hospitals	Antimicrobial consumption after introduction of an antimicrobial stewardship program defined as a program able to continuously monitor use with the support of a dedicated team in some hospitals	Evaluation of days of therapy per 1,000 patient-days	Decline in average antibiotic use in hospitals with antimicrobial stewardship program of 11 % vs 8 % in those without the program
Filkelstein et al., 2008 [40]	The community (16 non-overlapping communities)	Guideline dissemination, small-group education, frequent updates, education material for paediatricians and parents	Consumption of antibiotics	The intervention had no effect among children aged 3–23 months but was associated with a 4 % and 7 % decrease in antibiotic prescriptions in those aged 24–47 months and 48–71 months, respectively.
Gerber et al., 2013 [41]	The community (162 clinicians)	One 1-h on-site education session followed by one year of personalized quarterly audits and feedback on prescriptions for respiratory infections or usual practice in a group of enrolled individuals	Consumption of antibiotics	Broad spectrum antibiotic prescriptions decreased from 27 % to 14 % among intervention practices and from 28 % to 23 % in controls. Off-guideline prescriptions decreased from 16 % to 4 % among intervention practices compared with a decrease from 17 % to 16 % in controls.

Agwu et al. evaluated a World Wide Web-based antimicrobial restriction program at a 175-bed, tertiary care paediatric teaching hospital [36]. The program provided automated clinical decision support, facilitated approval, and enhanced real-time communication among prescribers, pharmacists, and paediatric infectious disease fellows. After implementation of the program, there was a $370,069 reduction in projected annual costs associated with restricted antimicrobial use and an 11.6 % reduction in the number of dispensed doses. User satisfaction increased from 22–68 % and from 13–69 % among prescribers and pharmacists, respectively. There were 21 % and 32 % reductions in the number of prescriber reports of missed and delayed doses, respectively, and there was a 37 % reduction in the number of pharmacist reports of delayed approvals [36].

Metjian et al. performed a prospective observational study in which data were collected on clinician's requests for targeted antibiotics and the interventions made by an AS program [37]. During the 4-month study period, calls were placed to the AS program for 652 patients. Forty-five percent of those calls required an intervention by the AS program. These interventions included targeting the known or suspected pathogens (20 %), consultation (43 %), optimizing the antimicrobial treatment (33 %), and stopping the antimicrobial treatment (4 %). Three of the 84 (3.5 %) patients recommended to receive an alternative therapy developed an infection not covered by the AS recommendations or the antimicrobial initially requested by the clinician [37].

Di Pentima et al. reported the results of an AS program carried out in a paediatric teaching hospital in Tennessee, USA [38]. An automated report of antimicrobials prescribed, doses, patient demographics, and microbiology data were generated and reviewed by an infectious disease pharmacist and a paediatric infectious disease physician. Antimicrobial use, expressed as the number of doses administered per 1,000 patient-days, was measured 3 years before and 3 years after the implementation of the program. Total antimicrobial use peaked at 3,089 doses administered per 1,000 patient-days per year in the last period before implementation of the program and decreased to 1,904 doses administered per 1,000 patient-days per year during the last post-intervention period [38]. Targeted-antimicrobial use declined from 1,250 to 988 doses administered per 1,000 patient-days per year. Nontargeted-antimicrobial use declined from 1,839 to 916 doses administered per 1,000 patient-days per year. Rates of antimicrobial resistance to broad-spectrum antimicrobials among the most common Gram-negative bacilli remained low and stable over time [38].

However, all of these studies were single-centre studies and were carried out over a limited period of time. For these reasons, they can be criticized because they are subject to multiple biases, including publication bias, a failure to consider secular trends in antibiotic use and a lack of comparisons with antibiotic prescriptions in similar hospitals without AS programs. The study by Hersh et al. overcame these limits, confirming the effectiveness of AS programs in children and the need for their systematic use in all paediatric hospitals [39]. These authors compared antibiotic prescription rates in a group of nine paediatric hospitals with formal AS programs to corresponding rates in a group of 22 control hospitals without formal AS programs. The impact of AS on antibiotic prescriptions was measured by days of therapy/1,000 patient-days during the period from 2004–2012 before and after the release of 2007 IDSA guidelines for developing AS programs. Antibiotic use was compared for all antibacterials and for a select subset (i.e., vancomycin, carbapenems, linezolid). In comparison with the decline observed in those years preceding the guidelines, there was a larger post-guideline decline in average antibiotic use in hospitals with AS programs than in hospitals without them (11.0 % vs 8.0 %, respectively; p = 0.04). Because the decline in antibiotic use was lower in the pre-implementation period (5.7 %), it was concluded that these results provide evidence that the reduction was greater than the concurrent secular trend and supported the value of AS programs.

Outpatient AS interventions are uncommon. However, the available data seem to indicate that AS can be effective in reducing antibiotic misuse in community settings as well, although the results are less impressive than those obtained in hospitals. From 1998–2003 Finkelstein et al. conducted a controlled, community-level, cluster-randomized trial in 16 non-overlapping Massachusetts communities [40]. During the winter periods of these 3 years, some of these communities implemented a physician behaviour-change strategy that included guideline dissemination, small-group education, frequent updates and educational materials, and feedback on prescriptions. Moreover, parents of children living in these communities received educational materials by mail and in primary care practices, pharmacies, and child care settings. No intervention was planned in the remaining communities. The number of antibiotics dispensed per person-year of observation among children aged 3 to <72 months who resided in the study communities was evaluated. A substantial downward trend in antibiotic prescriptions, even in the absence of the intervention, was observed. The intervention had no additional effect among children aged 3 to <24 months but was responsible for a 4.2 % decrease in antibiotic prescriptions among those aged 24 to <48 months and a 6.7 % decrease among those aged 48 to <72 months [40]. The intervention effect was greater for broad-spectrum agents.

More recently, Gerber et al. performed an outpatient AS interventional trial that involved 162 clinicians and consisted of a 1-h on-site clinician education session followed by one year of personalized quarterly audits of and feedback on prescriptions for acute respiratory tract infections or usual practice [41]. Broad-spectrum antibiotic prescriptions decreased from 26.8 % to 14.3 % among intervention practices and from 28.4 % to 22.6 % in control settings (p = 0.01). Off-guideline prescriptions for children with pneumonia decreased from 15.7 % to 4.2 % among intervention practices compared with a decrease from 17.1 % to 16.3 % in control practices (p < 0.001). However, no significant differences were observed for acute rhinosinusitis; off-guideline prescriptions for this condition declined from 38.9 % to 18.8 % in intervention practices and from 40.0 % to 33.9 % in control facilities (p = 0.12). Off-guideline prescriptions were uncommon at baseline and changed little for streptococcal pharyngitis (intervention, from 4.4 % to 3.4 %; control, from 5.6 % to 3.5 %; p = 0.82) and for viral infections (intervention, from 7.9 % to 7.7 %; control, from 6.4 % to 4.5 %; p = 0.93) [41].

One of the most successful interventions to reduce antibiotic prescriptions among children in outpatient settings has been performed in Sweden [42]. Since 1992–2014, the total consumption of antibiotics has decreased by 41 %. The greatest decrease during these years has been in the 0–4 year age group, where sales decreased by 75 %, from 1,328 in 1992 to 328 prescriptions per 1,000 inhabitants in 2014.

### Barriers to the development and implementation of antimicrobial stewardship programs

Despite a growing evidence base supporting the value of paediatric AS programs, a number of well-recognized barriers hinder their development and growth. A lack of knowledge regarding the fundamental rules on which antibiotic prescriptions are based remains one of the most important problems in the implementation of AS programs. A recent study by Bowes et al. highlighted several challenges that paediatric practitioners face with respect to the knowledge of and approaches to prescribing antimicrobials [43]. In particular, these authors reported that the majority of physicians included in a survey they carried out in a Canadian teaching hospital did not receive any formal education regarding the prescribing of antimicrobials and antimicrobial stewardship in the previous year. Moreover, both the trainees and the staff had modest knowledge of factors that would increase the risk of resistance, and less than 20 % of them had correct knowledge of local resistance patterns for common bacteria [43].

All of these findings provide evidence that educational programs specifically devoted to illustrate the rules for rational use of antibiotics are needed before and during AS program implementation. Guidelines and protocols can be useful in this regard, but to be most effective, these should be shared between all specialists directly or indirectly involved in the prescription of antibiotics, including pharmacists, microbiologists, and infectious disease specialists. Shared suggestions for antibiotic administration can permit a facility to overcome the risk of any new intervention being perceived by front-line physicians as interfering with their routine practice and being questioned. Unfortunately, creation of a team specifically dedicated to educate providers and monitor antibiotic use to correct possible mistakes is not always possible because it requires substantial financial support. A lack of funds can cause difficulties in the implementation of an AS program. Additionally, a lack of technical resources may further limit the implementation and effectiveness of AS programs. Daily surveillance of antibiotic prescriptions and control of the concordance between actual and suggested uses, including evaluations of dosing and length of administration, are largely facilitated by the availability of computerized programs. Availability of hardware and software needed for monitoring first requires financial support and administrative consensus, which is not easily obtained, even though cost saving is routinely achieved by reducing unnecessary antibiotic use and decreases in the pharmacy budget usually cover the expenses for AS program implementation within a few months [44].

### Future areas of research

As recently indicated by the updated IDSA guidelines for implementing an AS program [45], passive didactic education and facility-specific clinical practice guidelines are only useful complement of a global AS intervention and have to be associated with control measures of antibiotic use that can modify the debatable prescriptions. The importance of these interventions is clearly evidenced by a recent evaluation of the antibiotic use in paediatrics carried out in Italy [46]. In this country, since long time several initiatives to improve paediatrician’s education on antibiotic use for the most common paediatric infections have been made [47–49], but no control of guidelines impact with feedback to clinicians assessing the appropriateness of continuing antibiotic therapy has been performed. The recent evaluation of antibiotic prescriptions in several Italian pediatric departments has shown an over-use of broad-spectrum antibiotics such as third generation cephaloporins and carbapenems for both prophylaxis and treatment [46]. Future areas of research should try to link antimicrobial resistance to antibiotic prescriptions analysing the impact of AS programes in paediatric care.

## Conclusions

Among adult patients, the successful implementation of AS strategies have had a significant impact on reducing targeted- and nontargeted-antimicrobial use, improving quality of care of hospitalized patients and preventing the emergence of resistance. Similar results have also been reported in paediatric patients, although there has been significantly less widespread implementation of AS programs among children. However, not all of the problems related to implementation of AS programs in paediatric settings are solved. The most important remaining problems involve educating paediatricians, creating a multidisciplinary interprofessional AS team able to prepare guidelines, monitoring antibiotic prescriptions and defining corrective measures, and the availability of administrative consensuses with adequate financial support. Additionally, the problem of optimizing the duration of AS programs remains unsolved. It is not clear whether such programs must be maintained continuously or can instead be implemented until rationalization of antibiotic use is obtained and re-initiated when significant variations in antibiotic use are needed. Some reports, including the recent experience of Gerber et al. in a community setting [50], seem to indicate that AS program discontinuation is dangerous because without monitoring, antibiotic use tends to revert to the initial levels. Further studies are needed to solve the above mentioned problems. However, considering that rationalization of antibiotic misuse and abuse is the basis for reducing emergence of bacterial resistance, all efforts must be made to develop multidisciplinary paediatric AS programs.

## Abbreviations

AS: Antimicrobial stewardship
